# Prediction of major intravascular hemolysis during pulsed electric field ablation of atrial fibrillation using a pentaspline catheter

**DOI:** 10.1111/jce.16468

**Published:** 2024-10-13

**Authors:** Predrag Stojadinović, Nicoletta Ventrella, Hana Alfredová, Dan Wichterle, Petr Peichl, Robert Čihák, Vanda Filová Ing, Eva Borišincová, Petr Štiavnický, Jana Hašková, Janka Franeková, Josef Kautzner

**Affiliations:** ^1^ Department of Cardiology Institute for Clinical and Experimental Medicine Prague Czechia; ^2^ Institute of Physiology Charles University Medical School I Prague Czechia; ^3^ Department of Clinical Sciences and Community Health, Cardiovascular Section University of Milan Milan Italy; ^4^ Department of Biochemistry Institute for Clinical and Experimental Medicine Prague Czechia

**Keywords:** acute kidney injury, atrial fibrillation, hemolysis, pulsed field ablation

## Abstract

**Introduction:**

Pulsed electric field (PEF) has emerged as a promising energy source for catheter ablation of atrial fibrillation (AF). However, data regarding the in‐vivo effect of PEF energy on erythrocytes during AF ablation procedures are scarce. This study aimed to quantify the impact of PEF energy on erythrocyte damage during AF ablation by assessing specific hemolytic biomarkers.

**Methods:**

A total of 60 patients (age: 68 years, males: 72%, serum creatinine: 91 µmol/L) with AF underwent catheter ablation of AF using PEF energy delivered by a multipolar pentaspline Farawave catheter (Farapulse, Boston Scientific, Inc.). Ablation beyond pulmonary vein isolation was performed at the operator's discretion. Peripheral venous blood was sampled for assessing the plasma levels of free hemoglobin (fHb), direct (conjugated) bilirubin, lactate dehydrogenase (LDH), and creatinine before, immediately after the ablation, and on the next day.

**Results:**

Following the PEF ablation with duration of [median (interquartile range)] 75 (58, 95) min, with 74 (52, 92) applications and PVI only in 27% of patients, fHb, LDH, and direct bilirubin significantly increased, from 40 (18, 65) to 493 (327, 848) mg/L, from 3.1 (2.6, 3.6) to 6.8 (5.0, 7.9) µkat/L, and from 12 (9, 17) to 28 (16, 44) µmol/L, respectively (all *p* < .0001). A strong linear correlation was found between the peak fHb and the number of PEF applications (*R* = 0.81, *p* < .001). The major hemolysis (defined as fHb >500 mg/L) was predicted by the number of PEF applications with the corresponding area under the receiver operating characteristic curve of 0.934. The optimum cut‐off value of >74 PEF applications predicted the major hemolysis with 89% sensitivity and 87% specificity.

**Conclusion:**

Catheter ablation of AF using PEF energy delivered from a pentaspline catheter is associated with significant intravascular hemolysis. More than 74 PEF applications frequently resulted in major hemolysis. However, the critical amount of PEF energy that may cause kidney injury in susceptible patients remains to be investigated.

**Abbreviations**
AF: atrial fibrillation
AKI: acute kidney injury
fHb: free hemoglobin
Hb: hemoglobin
IQR: interquartile range
LDH: lactate dehydrogenase
PEF: pulsed electric field
PFA: pulsed field ablation
PVI: pulmonary vein isolation
ROC: receiver operating characteristics

## 1 INTRODUCTION

In recent years, pulsed electric field (PEF) has emerged as a promising energy modality for catheter ablation of atrial fibrillation (AF), offering a viable alternative to thermal methods such as radiofrequency or cryoenergy‐based ablation. PEF ablation (PFA) harnesses microsecond‐scale, high‐voltage electric fields to induce irreversible electroporation and destabilization of cell membranes, ultimately leading to cellular necrosis.^1^ Its noteworthy tissue specificity permits targeted ablation of myocardial tissue while minimizing inadvertent damage to neighboring structures including the esophagus, and phrenic nerve.^1^, ^2^

However, a concern arises regarding the potential hemolytic effects of PEF energy on red blood cells. Available evidence suggests that hemolysis ensues through a field‐dependent process initiated by pore formation and subsequent ion leakage, inducing osmotic imbalance and erythrocyte swelling, culminating in colloid osmotic hemolysis.^3^ Hemolysis following catheter ablation may reach clinical significance due to the release of hemoglobin and free heme, which can disrupt tubular barriers and induce oxidative cell damage, potentially predisposing individuals to acute kidney injury.^4^

Nevertheless, there is a paucity of data regarding the in vivo impact of PEF energy on erythrocytes during AF ablation procedures.^5^, ^6^ Hence, our objective was to quantify this by evaluating specific hemolytic biomarkers and identifying potential predictors of major hemolysis after catheter ablation with a pentaspline catheter.

## 2 METHODS

### 2.1 Study population

A study included 60 consecutive patients undergoing PFA for paroxysmal or persistent AF were included in the analysis. All individuals received pulmonary vein isolation (PVI) using PEF energy delivered by a multipolar pentaspline Farawave catheter (Farapulse, Boston Scientific, Inc.). Additional ablations of the left atrial posterior wall, roof of the left atrium, mitral isthmus or cavo‐tricuspid isthmus were performed at the operator's discretion. All patients signed informed consent with the procedure. The study protocol was approved by the local institutional review board.

### 2.2 Ablation procedure

Procedures were conducted under general anesthesia using volatile agent sevoflurane or under deep sedation using fentanyl and propofol infusion. Heparin was administered before the transseptal puncture to achieve activated clotting time between 300 and 350 s, with vigilant monitoring at 15 to 30‐min intervals. The procedure was performed with fluoroscopic navigation and guided by intracardiac echocardiography. The left atrium was accessed by a 13‐F deflectable transseptal sheath. Pulmonary vein isolation was accomplished using the FARAPULSE™ Pulsed Field Ablation System (Boston Scientific) with four applications each in “flower” and “basket” configurations (with rotation between each pair of lesions), totaling eight applications per vein. These were frequently complemented with additional ablations at wider pulmonary vein antra. Confirmation of isolation relied on electrograms recorded from the multispline catheter and loss of atrial capture by pacing inside the vein. Anatomical and/or electrogram‐guided ablation of other left‐ and/or right‐atrial sites has been conducted at the discretion of the operator depending on current arrhythmia manifestation/inducibility, and substrate, activation or entrainment mapping. These lesions were also created by ablation catheter in the “flower” and/or “basket” configurations. Before each application, the position of the pentaspline and the proper contact between the catheter and tissue were assessed using intracardiac echocardiography. Peripheral venous blood samples were collected from all patients to assess plasma levels of free hemoglobin (fHb), direct (conjugated) bilirubin, lactate dehydrogenase (LDH), creatinine, and total hemoglobin (Hb) at baseline, immediately after the ablation, and on the next day (usually 18–21 h after the procedure. Patient data were prospectively gathered in an institutional review board‐approved database. The definition of the major hemolysis as fHb >500 mg/L was derived from the official guidelines provided by the Extracorporeal Life Support Organization (ELSO) for managing patients with mechanical circulatory support, where hemolysis is a significant concern.^7^, ^8^ Procedures were carried out by four operators, all of whom had performed more than 50 procedures.

### 2.3 Statistical analysis

All statistical analyses were conducted in R (http://www.R-project.org). Continuous variables are described as medians with interquartile range (IQR). Categorical variables are presented as counts (percentages). Study groups were compared using the Wilcoxon test, Kruskal‐Wallis, Person's chi‐squared, and Fisher exact test as appropriate. A *p* < .05 was considered significant throughout the study. Receiver operating characteristic (ROC) analysis was performed to evaluate the threshold value of the total number of applications in differentiating the major from mild hemolysis. Optimal cut‐off values were identified by the Youden method.

## 3 RESULTS

**Figure 1:**
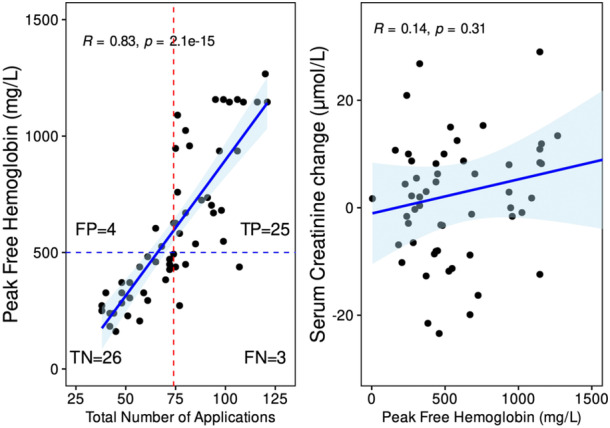
Left panel: Scatterplot illustrating the relationship between the total number of pulsed electric field (PEF) applications and the peak free hemoglobin (left panel). Dashed lines indicate the optimum cut‐off number of PEF applications (in red) to predict the major hemolysis (in blue). Right panel: Scatterplot illustrating the relationship between the total number of PEF applications and the serum creatinine change. FN, false negatives; FP, false positives, TN, true negatives; TP, true positives.

**Figure 2:**
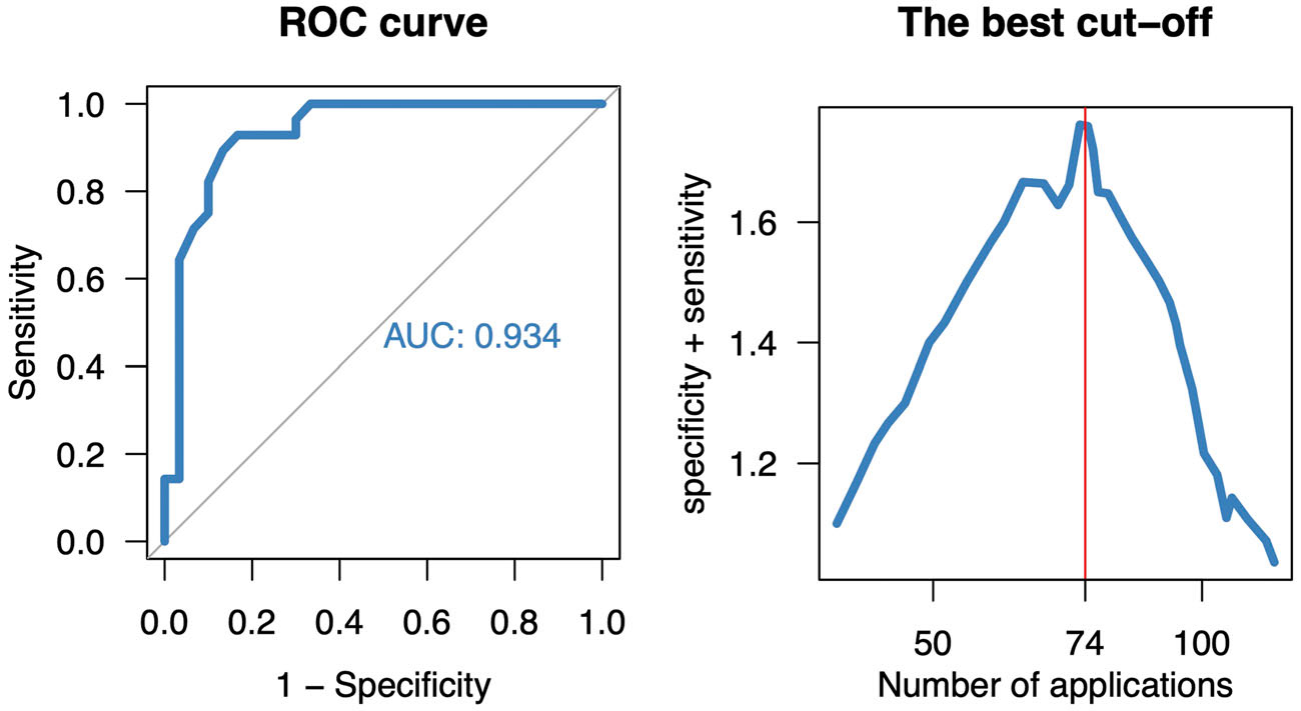
Left panel: Receiver operating characteristic curve (ROC) for predicting major hemolysis by the total number of pulsed electric field (PEF) applications. Right panel: The optimal cut‐off value is based on the highest sum of sensitivity and specificity (Youden method). AUC, area under the curve.

**Figure 3:**
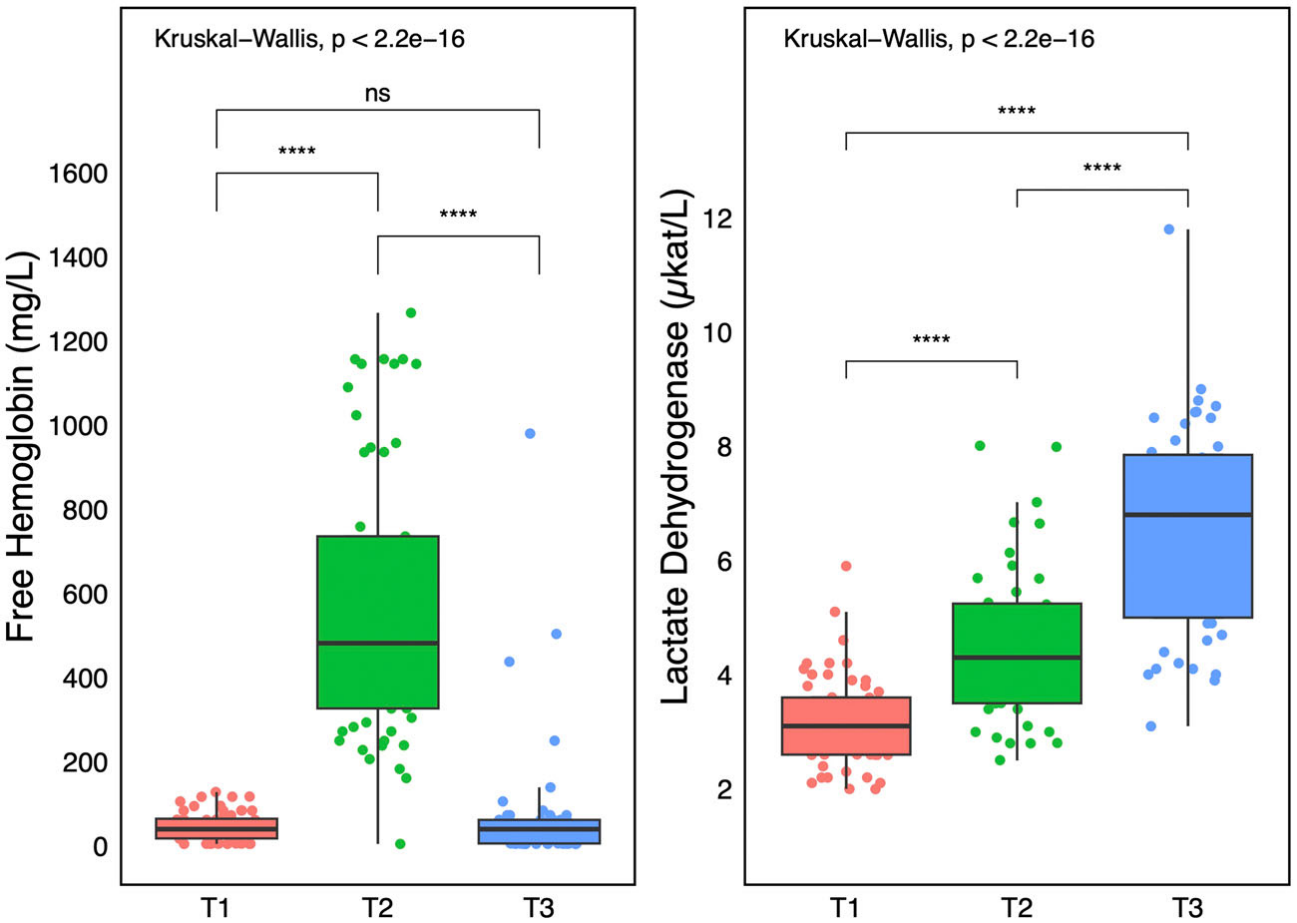
Time–course of free hemoglobin and lactate dehydrogenase. T1— before the procedure; T2—at the end of the procedure, T3—the next day (usually 18–21 h after the procedure). Statistical significance markers—ns: *p* ≥ .05; *p < .05; **p < .01; ***p < .001; ****p < .0001.

All enrolled patients completed the study protocol without complications, including acute kidney injury (AKI). The baseline patient and procedural characteristics are summarized in Tables 1 and 2. Following the PEF ablation with duration of [median (IQR)] 75 [58,95] min, with 74 (52, 92) applications, and PVI only in 27% of patients, fHb increased from 40 (18, 65) to 493 (327, 848) mg/L, LDH increased from 3.1 (2.6, 3.6) to 6.8 (5.0, 7.9) µkat/L, and direct bilirubin increased from 12 (9, 17) to 28 (16, 44) µmol/L (all *p* < .0001). A total of 29 patients had fHb ≥500 mg/L with a median (IQR) of 493 (327, 848) mg/L (Table 3, Figure 1). Figure 1 depicts the significant linear correlation between the peak fHb and the total number of PEF applications (*R* = 0.81, *p* < .001). The major hemolysis was predicted by the number of PEF applications with the corresponding area under the ROC curve of .934. The optimum cut‐off value of 74 PEF applications predicted the major hemolysis with 89% sensitivity and 87% specificity (Figure 2). The time‐line changes of fHb and LDH are shown in Figure 3. The major hemolysis occurred in one out of 15 patients (6,7%) who underwent PVI only and 28 out of 45 patients (62%) who had additional application beyond the PVI (*p* = .0002 by Fisher exact test). No significant change in creatinine level was observed, and no significant correlation was found between the peak fHb and serum creatinine change (Figure 1). However, there was a significant change in total Hb, urea, and total bilirubin after the ablation procedures.

**Table 1:** Baseline characteristics (*N* = 60).

Age (years)	68 (62, 72)
Male	43 (72%)
Arterial hypertension	53 (88%)
Diabetes mellitus	6 (10%)
Coronary artery disease	7 (12%)
Transient ischemic attack or stroke	2 (3.3%)
Body mass index (kg/m^2^)	28.8 (25.6, 32.3)
Paroxysmal atrial fibrillation	31 (51.7%)
Atrial fibrillation history (months)	37 (14, 91)
Left ventricular end‐diastolic diameter (mm)	53 (50, 56)
Left ventricular ejection fraction (%)	60 (58, 60)
Left atrial diameter (mm)	43 (39, 48)
Left atrial volume index (mL/m^2^)	42 (34, 49)

*Note*: Values are *n* (%) or medians (IQR).

**Table 2:** Procedural characteristics (*N* = 60).

Sinus rhythm on admission	42 (71%)
Duration (min)	75 (58, 95)
Number of applications	74 (52, 92)
Pulmonary vein isolation only	16 (27%)
Posterior wall/roof ablation	39 (65%)
Cavo‐tricuspid isthmus ablation	29 (48.3%)
Anterior/lateral mitral isthmus ablation	25 (41.7%)
Number of operators	4
General anesthesia	41 (68%)
Deep analgosedation	19 (32%)
Systolic blood pressure (mmHg)	97 (85, 117)
Diastolic blood pressure (mmHg)	57 (53, 70)
Right atrial pressure (mmHg)	7 (5, 8)
Left atrial pressure (mmHg)	8 (6, 10)

*Note*: Values are *n* (%) or medians (IQR).

**Table 3:** Laboratory data (*N* = 60).

	Before procedure	After procedure	*p* Value^a^
Sodium (mmol/L)	141 (139, 142)	139 (137, 140)	< .001
Potassium (mmol/L)	4.3 (4.2, 4.5)	4 (3.9, 4.2)	< .001
Chloride (mmol/L)	107 (105, 108)	107 (106, 108)	> .9
Urea (mmol/L)	6.3 (5.4, 7.4)	7.2 (6.3, 9.2)	.002
Creatinine (μmol/L)	91 (80, 102)	95 (84, 108)	.4
Hemoglobin (g/L)	146 (137, 159)	136 (122, 145)	< .001
Free hemoglobin (mg/L)	40 (18, 65)	493 (327, 848)^b^	< .001
Lactate dehydrogenase (µkat/L)	3.1 (2.6, 3.6)	6.8 (5, 7.9)	< .001
Direct bilirubin (µmol/L)	12 (9, 17)	28 (16, 44)	< .001

*Note*: Values are median (IQR).

^a^ Wilcoxon rank sum test.

^b^ Peak level at the end of the procedure.

## 4 DISCUSSION

We validated a tight relationship between the rise in fHb and the number of PFA applications in human AF ablation procedures. We found that patients with more than 74 PEF applications from the pentaspline ablation catheter are at risk of having major hemolysis, defined as peak fHb of more than 500 mg/dL. Despite the significant proportion of patients with major hemolysis, none of the patients exhibited clinically significant impairment of renal function attributable to intravascular hemolysis.

Previous studies have elucidated that applying an electric field of 1.6–2.5 kV/cm to human erythrocytes generates a transmembrane potential of approximately 1.0 V, resulting in electroporation occurring within microseconds and leading subsequently to hemolysis.^3^, ^9^ PFA entails the application of ultra‐rapid electrical pulses ranging from microseconds to nanoseconds to generate robust electrical fields, thereby inducing irreversible nanoscale pore formation and ultimately causing cell death. Given that the electrical field magnitude necessary to elicit irreversible electroporation is approximately 700 V/cm for the myocardium,^10^ the electrodes of the ablation catheter for PFA energy delivery are designed to administer a voltage between 900 and 2500 V.^11^ With each PFA application, electric fields surpassing 2500 V/cm can be attained near the electrodes. Although minimal hemolysis may occur with each application, extensive ablation involving many applications could proportionally escalate the degree of hemolysis until a clinical manifestation is evident.

Current literature provides limited evidence regarding the occurrence of hemolysis following catheter ablation of AF using PEF. Neither the MANIFEST^12^ nor the EUPORIA^13^ registries reported this complication. This lack of reporting could be attributed to the prevalence of procedures with pulmonary vein isolation only in both registries, thereby limiting the risk of extensive ablations.

Similarly, the recent ADVENT trial,^1^ which randomized patients with drug‐refractory paroxysmal AF in a 1:1 ratio to PFA or conventional radiofrequency or cryoballoon ablation, did not report any instances of hemolysis. However, a recent presentation by Reddy and Ekanem at the AHA,^14^ with supplementary data from the MANIFEST‐17K study, revealed cases of acute kidney injury necessitating dialysis secondary to hemolysis. In addition, two prospective studies investigated the incidence of hemolysis following PFA and its impact on renal function. Venier et al.,^5^ after having reported two cases of acute kidney injury associated with severe hemolysis, enrolled a cohort of 68 consecutive patients undergoing PFA for AF (the median number of applications was 64). Of these patients, 19 (28%) exhibited significantly depleted haptoglobin levels after ablation, indicating significant hemolysis. The study observed a significant inverse correlation between haptoglobin levels and the total number of applications, with over 70 applications demonstrating the optimal sensitivity and specificity in predicting haptoglobin depletion. This is in agreement with our threshold of 74 applications. Accordingly, the original two patients received 174 and 126 PEF applications. Despite the not negligible incidence of hemolysis in their cohort, the median creatinine level did not significantly increase after the procedure. Similarly, Mohanty et al.^6^ divided 103 consecutive patients with AF undergoing PFA into two groups based on post‐procedural hydration. Group 1 (28 patients), consisting of patients who did not receive immediate hydration, showed a higher incidence of hemoglobinuria within 24 h postablation than group 2 (75 patients), which received post‐procedural fluid infusion. The mean postablation serum creatinine was significantly elevated compared to the baseline value in those patients from group 1 who experienced hemoglobinuria. On the other hand, in patients from group 2, no significant changes in creatinine were recorded. Furthermore, the multivariable analysis identified both hydration status and the number of PFA applications as independent predictors of post‐procedural acute kidney injury. Another study by Nies et al.^15^ demonstrated in vitro that good catheter‐tissue contact during single‐shot PEF ablation may reduce hemolysis by minimizing blood pool exposure to the electrical field.

Our findings indicate a linear relationship between hemolysis occurrence and the number of PFA applications. Therefore, patients' comorbidities and the potential for procedural hemolysis should be considered when planning ablations beyond pulmonary vein isolation. A recently published study from Jordan et al.^16^ reported a very low incidence of AKI after PEF pulmonary vein isolation, even lower than after ablation utilizing thermal energies. This means that AKI following catheter ablation cannot be explained solely by the hemolysis.

The cut‐off value of 74 applications is merely a threshold for predicting significant hemolysis using laboratory markers. It does not account for other variables influencing renal functions, such as anesthesia‐induced hypotension, hydration status, periprocedural use of contrast agents, and numerous patient‐specific variables.

Although evidence regarding the association between hemolysis and renal damage remains limited, monitoring renal function parameters in the post‐procedural period represents a prudent clinical practice. Besides the potential renal toxicity, previous studies demonstrated a significant risk of injury to other organs, including the heart, brain, and microvascular systems.^17^ Therefore, preventative hydration before and/or after the procedure should be considered in case of extensive PEF ablation to mitigate the adverse effects of cell‐free hemoglobin toxicity.

### 4.1 Study limitations

This was a merely technical study showing the impact of the number of PEF energy applications on the magnitude of intravascular hemolysis. All patients had normal left ventricular systolic function and renal function, and none received a contrast agent before or during the procedure. Data regarding the long‐term dynamics of renal function after the ablation are also missing. Therefore, the study could underestimate the risk of AKI in the general population of patients who are undergoing the PFA for AF. Additionally, the plasma creatinine level has low sensitivity in identifying early or mild forms of renal damage. We have not collected information about the proportion of PEF applications with the catheter in “basket” configuration outside the pulmonary veins that may enhance the hemolysis because of noncontacting catheter splines.

It is important to emphasize that our results refer to one specific PFA technology – the Farapulse system, which uses the pentaspline catheter with interchangeable shape and size. Other PFA platforms use different power and configurations of the PF energy, as well as specific catheter designs. The amount of PEF delivered to the blood and the risk of hemolysis may differ.

## 5 CONCLUSIONS

Catheter ablation of AF using PEF energy delivered from a specific pentaspline catheter is associated with significant intravascular hemolysis that is linearly related to the total number of PEF applications. However, the critical amount of PEF energy that may cause AKI in susceptible patients remains to be investigated separately for each PFA technology.

## AUTHOR CONTRIBUTIONS

1) Substantial contributions to the conception and design or the acquisition, analysis, or interpretation of the data: Nicoletta Ventrella, Predrag Stojadinović, Dan Wichterle, Petr Peichl, Hana Alfredová Ing, Vanda Filová, Robert Čihák, Petr Štiavnický, Jana Hašková, Eva Borišincová, Janka Franeková, and Josef Kautzner.
2) Substantial contributions to the drafting of the articles or critical revision for important intellectual content: Nicoletta Ventrella, Predrag Stojadinović, Dan Wichterle, and Josef Kautzner.
3) Final approval of the version to be published: Josef Kautzner.
4) Agreement to be accountable for all aspects of the work in ensuring that questions related to the accuracy or integrity of any part of the article are appropriately investigated and resolved: Nicoletta Ventrella, and Predrag Stojadinović.

## Data Availability

The data that support the findings of this study are available from the corresponding author upon reasonable request.
